# Comparative Diagnostic Performance of Copeptin After Hypertonic Saline Infusion Versus Water Deprivation Test in Pediatric Patients with Polyuria–Polydipsia Syndrome

**DOI:** 10.3390/ijms26125449

**Published:** 2025-06-06

**Authors:** Diana-Andreea Ciortea, Carmen Loredana Petrea (Cliveți), Iolanda Cristina Vivisenco, Sorin Ion Berbece, Gabriela Gurău, Mădălina Nicoleta Matei, Aurel Nechita

**Affiliations:** 1 Faculty of Medicine and Pharmacy, “Dunarea de Jos” University of Galati, 800201 Galati, Romania; diana.ciortea@ugal.ro (D.-A.C.); carmen.petrea@ugal.ro (C.L.P.); aurel.nechita@ugal.ro (A.N.); 2 “Maria Sklodowska Curie” Emergency Clinical Hospital for Children, 041451 Bucharest, Romania; 3 “Sf Ioan” Emergency Clinical Hospital for Children, 800487 Galati, Romania; 4 Faculty of Medicine, “Carol Davila” University of Medicine and Pharmacy, 030167 Bucharest, Romania; cristina.vivisenco@umfcd.ro; 5 Department of Pediatrics, Emergency Clinical Hospital for Children “Grigore Alexandrescu”, 011743 Bucharest, Romania

**Keywords:** copeptin, central diabetes insipidus, nephrogenic diabetes insipidus, primary polydipsia, water deprivation test, arginin–vasopresin

## Abstract

Differentiating central diabetes insipidus (CDI), nephrogenic diabetes insipidus (NDI), and primary polydipsia (PP) in pediatric patients with polyuria–polydipsia syndrome (PPS) remains a clinical challenge. The water deprivation test (WDT) is the traditional gold standard; however, it is time-consuming, burdensome, and prone to equivocal results. Stimulated copeptin, a surrogate marker of vasopressin, has emerged as a promising diagnostic alternative. We conducted a prospective, observational, cross-sectional study involving 27 pediatric patients (ages 2–17) presenting with PPS. Each patient underwent a WDT with desmopressin and hypertonic saline infusion (3% NaCl) for stimulated copeptin testing. Diagnostic accuracy was assessed using clinical diagnoses as a reference. The WDT showed high accuracy with an area under the curve (AUC) of 0.97, and there was an increased optimal threshold of ≥14% urine osmolality after desmopressin acetate (1-deamino-8-D-arginine vasopressin, DDAVP) administration (sensitivity 88.9%, specificity 100%). Stimulated copeptin at a threshold of <6.5 pmol/L demonstrated 100% sensitivity and specificity (AUC = 1.00) for CDI versus PP. Basal copeptin ≥21.4 pmol/L accurately identified all NDI cases. The agreement between the WDT and copeptin was low (κ = 0.06, McNemar *p* = 0.021), suggesting that copeptin has greater specificity, particularly for borderline or partial CDI. These results support the use of stimulated copeptin as a first-line diagnostic tool in pediatric PPS, offering improved objectivity, tolerability, and diagnostic clarity compared with the WDT. Basal copeptin also demonstrated excellent performance in rapid noninvasive NDI identification.

## 1. Introduction

Polyuria–polydipsia syndrome (PPS) in pediatric patients is a rare but clinically important presentation that requires careful differentiation between central diabetes insipidus (CDI), nephrogenic diabetes insipidus (NDI), and primary polydipsia (PP). Accurate diagnosis is essential because these conditions have distinct underlying mechanisms, require tailored treatments, and differ in long-term outcomes. However, distinguishing between them is often challenging due to overlapping clinical features and variability in water intake and urine output in patients. Failure to accurately differentiate them can lead to unnecessary lifelong therapies, electrolytic imbalances, or delayed management, especially in children and adolescents [1,2].

The conventional diagnostic standard, especially in adult patients, is the water deprivation test (WDT) followed by desmopressin acetate or 1-deamino-8-D-arginine vasopressin (DDAVP) administration, which is a synthetic analog of the antidiuretic hormone. This test has long been used to assess the ability of the kidneys to concentrate urine in response to endogenous and exogenous antidiuretic hormone (ADH). Although the WDT remains the classical reference, it is laborious, time-consuming, and frequently produces inconclusive results, particularly in cases of partial CDI or PP [3,4]. In pediatric populations, the physical and emotional burden of prolonged dehydration often limits the feasibility of this approach [4].

Recent advances have shifted the focus toward more objective physiological biomarkers, such as copeptin, a stable C-terminal fragment of vasopressin prohormone that is released in equimolar amounts with AVP. Unlike vasopressin, copeptin can be reliably measured in serum using chemiluminescence- or immunoassay-based platforms and has emerged as a promising diagnostic tool in both adults and children with PPS [5,6]. Hypertonic saline infusion stimulates copeptin release proportionally to plasma osmolality, and thresholds such as <4.9 pmol/L and <6.5 pmol/L have demonstrated high sensitivity and specificity for CDI diagnosis in prospective studies [7,8]. Additionally, basal copeptin levels of ≥21.4 pmol/L are strongly suggestive of NDI and offer a fast, noninvasive screening method [8].

From a clinical perspective, copeptin testing offers several advantages: it is less stressful for pediatric patients, faster to perform, and generally better tolerated than prolonged and demanding water deprivation tests. Previous pediatric studies have shown that the hypertonic saline infusion test is both safe and well tolerated when conducted under appropriate monitoring conditions [9]. This biomarker-based approach provides a technical advantage, especially in pediatric patients, where compliance with multiple phases of the WDT can be difficult. Copeptin testing eliminates the need for urine osmolality measurement or prolonged fluid restriction, thus reducing risk, discomfort, and diagnostic variability, especially in young children and adolescents [3,10].

Despite increasing evidence in adults, pediatric data on copeptin remain limited. Our recent systematic review and meta-analysis (Ciortea et al., IJMS 2024 [11]) identified only 11 eligible studies assessing copeptin as a diagnostic biomarker in PPS, and only 9 of these were strictly pediatric studies, with small sample sizes and heterogeneous methods [11]. Moreover, head-to-head comparisons between stimulated copeptin and the WDT in the same pediatric population are extremely rare, significantly limiting the generalizability of the previous findings. These gaps highlight the pressing need for prospective pediatric studies using standardized protocols and validated diagnostic thresholds.

Further evidence from Winzeler et al. [1] and other recent reviews also emphasizes the need for pediatric validation of adult-derived thresholds and supports the benefits of copeptin-based protocols in minimizing patient burden [12,13,14].

The differential diagnosis of polyuria–polydipsia syndrome (PPS) in pediatric patients is particularly challenging because of frequent concomitant electrolyte disturbances such as hyponatremia or hypernatremia, which can obscure the underlying pathophysiological mechanisms and complicate the interpretation of diagnostic tests [9,15]. These electrolyte imbalances may arise independently or in the context of underlying inflammatory or infectious conditions, including multisystem inflammatory syndrome in children (MIS-C) or sepsis, both of which can affect antidiuretic hormone (ADH) secretion and water balance [16,17,18]. In our previous study (Ciortea et al., CIMB 2024 [15]), we demonstrated that patients with MIS-C may exhibit inappropriate AVP secretion via non-osmotic mechanisms, leading to dilutional hyponatremia and mimicking the features of PP or partial CDI [15]. Therefore, it is critical to thoroughly evaluate and rule out systemic inflammatory conditions prior to diagnostic testing, particularly for basal copeptin measurement, as inflammation-related AVP dysregulation may interfere with interpretation.

To date, there is a lack of prospective pediatric studies directly comparing stimulated copeptin testing and the traditional WDT using validated thresholds in the same patient cohort. This absence of evidence creates diagnostic uncertainty in pediatric PPS and justifies the need for robust comparative data.

To address this evidence gap, the primary objective of our study was to directly compare the diagnostic performance of copeptin after 3% hypertonic saline infusion with that of the traditional WDT in the same cohort of pediatric patients with PPS, using validated diagnostic thresholds and final clinical diagnosis as the reference standard.

The secondary objectives were to evaluate the feasibility and tolerability of both tests, the diagnostic utility of basal copeptin for identifying NDI, and the agreement between WDT-based and copeptin-based classifications using statistical concordance tests (Cohen’s kappa, McNemar’s test).

We hypothesized that stimulated copeptin measurement following hypertonic saline infusion would demonstrate superior diagnostic accuracy, reliability, and patient tolerability compared to the traditional WDT in pediatric patients with polyuria–polydipsia syndrome. This approach is expected to enhance diagnostic performance, particularly in cases where the WDT is poorly tolerated.

To our knowledge, this is one of the first prospective pediatric studies to conduct a direct head-to-head comparison between the WDT and copeptin-based protocols using standardized thresholds and objective statistical evaluation. These findings may help refine diagnostic approaches for pediatric PPS and offer a more practical alternative when the WDT is inconclusive or impractical [7,8,9].

## 2. Results

### 2.1. Patient Characteristics

A total of 27 pediatric patients (median age: 11.2 years, range: 2–17 years; 59% male) with polyuria–polydipsia syndrome (PPS) were included. All patients underwent both the water deprivation test (WDT) followed by desmopressin (DDAVP) administration and copeptin testing after 3% hypertonic saline infusion.

Final clinical diagnoses were established based on a composite of the test results and clinical evaluation. The diagnostic distribution included 10 cases of complete central diabetes insipidus (CDI), 3 partial CDI, 4 nephrogenic diabetes insipidus (NDI), and 7 primary polydipsia (PP). Three initially inconclusive cases after the WDT were clarified using copeptin results.

### 2.2. Diagnostic Accuracy of the Water Deprivation Test (WDT)

**Figure 1:**
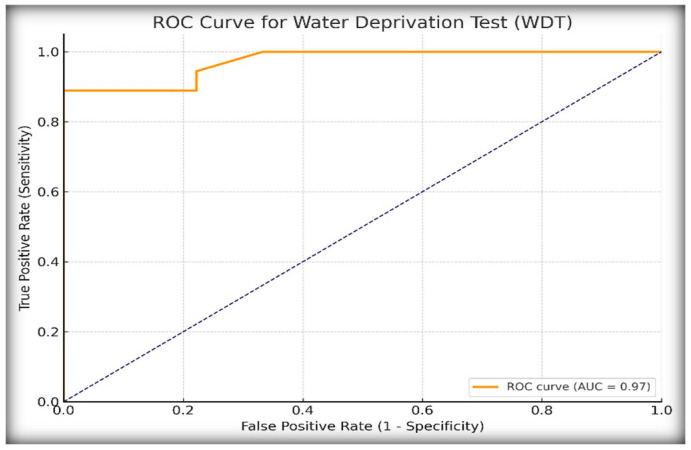
ROC curve for the water deprivation test (WDT) based on percentage increase in urine osmolality after desmopressin administration.

To assess the diagnostic performance of the WDT in distinguishing central diabetes insipidus (CDI) from primary polydipsia (PP), we analyzed the percentage increase in urine osmolality following desmopressin (DDAVP) administration. Receiver operating characteristic (ROC) curve analysis, as shown in Figure 1, identified a ≥14% increase in urine osmolality as the optimal diagnostic threshold based on the Youden index, which maximizes the sum of the sensitivity and specificity.

At this cut-off, the WDT demonstrated excellent diagnostic performance, as shown in Table 1, for identifying diabetes insipidus (CDI or NDI). The infinite positive Likelihood Ratio, LR+, was due to 100% specificity, meaning that a positive test result is decisively diagnostic of DI, and the low negative Likelihood Ratio, LR− (0.11), suggests that the WDT is also good at ruling out DI when the test is negative. These results confirm the high diagnostic utility of the WDT in identifying CDI in pediatric patients with polyuria–polydipsia syndrome, albeit with potential limitations in excluding partial or borderline cases.

### 2.3. Diagnostic Accuracy of Stimulated Copeptin

To evaluate the performance of copeptin following hypertonic saline infusion in differentiating central diabetes insipidus (CDI) from primary polydipsia (PP), we analyzed stimulated copeptin levels using two previously validated diagnostic thresholds: <4.9 pmol/L and <6.5 pmol/L. Table 2 presents the results. All patients clinically diagnosed with PP had copeptin values ≥6.5 pmol/L, while all CDI cases fell below this threshold, confirming the exceptional discriminatory power of the 6.5 pmol/L cut-off in this pediatric population.

**Figure 2:**
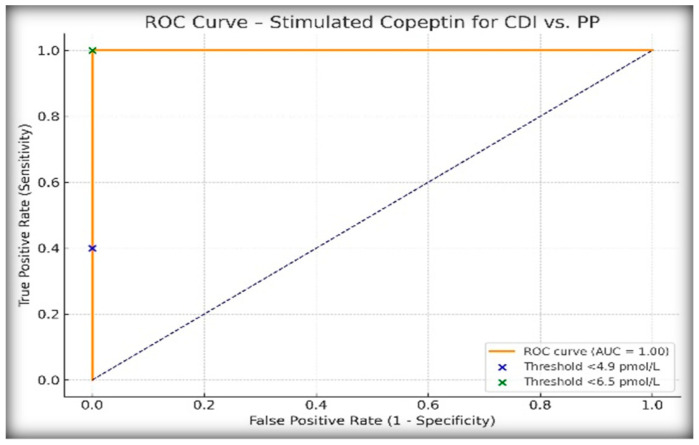
ROC curve for stimulated copeptin in differentiating central diabetes insipidus (CDI) from primary polydipsia (PP), with diagnostic thresholds <4.9 pmol/L and <6.5 pmol/L highlighted.

ROC curve analysis (Figure 2) demonstrated an AUC of 1.00, confirming the excellent discriminatory performance of stimulated copeptin for differentiating CDI from PP in our cohort. The positive predictive value (PPV) was 100% at the 6.5 pmol/L threshold, which reflects the complete diagnostic separation observed in our population and further supports the utility of this cut-off in clinical practice.

### 2.4. Diagnostic Utility of Basal Copeptin for NDI

Basal (unstimulated) copeptin concentrations were evaluated using a threshold of ≥21.4 pmol/L, which was previously validated for identifying nephrogenic diabetes insipidus (NDI). In our cohort, three patients had basal copeptin levels above this threshold. All three patients were clinically diagnosed with NDI based on their complete biochemical and clinical profiles. This basal copeptin value correctly identified NDI cases, with no overlap observed in the CDI and PP groups. This threshold yielded 100% sensitivity, specificity, PPV, and NPV, which is consistent with published reference values.

One patient initially classified as having partial NDI based on the WDT criteria did not meet the copeptin-based diagnostic thresholds, suggesting a potential misclassification or a borderline physiological profile. Stimulated copeptin levels in patients with confirmed NDI were markedly elevated (often exceeding 30 pmol/L), further reinforcing the diagnosis. None of the patients with CDI or PP exceeded the basal copeptin threshold.

**Figure 3:**
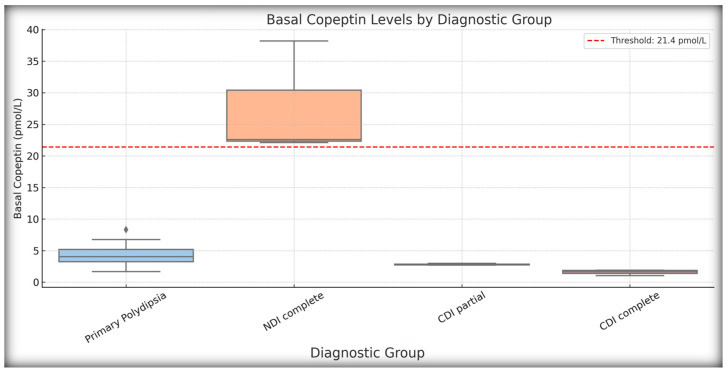
Basal copeptin levels by diagnostic group. Boxplot showing basal (unstimulated) copeptin concentrations across the main diagnostic categories: central diabetes insipidus (complete and partial), nephrogenic diabetes insipidus (NDI complete and partial), and primary polydipsia (PP). Each box represents the interquartile range (IQR), with the median marked by a horizontal line; whiskers extend to the minimum and maximum values, excluding outliers. The dashed red line at 21.4 pmol/L indicates the diagnostic threshold of NDI. All patients with basal copeptin ≥21.4 pmol/L were clinically confirmed as having NDI. Patients with CDI (complete and partial) exhibited uniformly low copeptin levels (<6.5 pmol/L), whereas PP cases showed intermediate values. This pattern supports the diagnostic utility of basal copeptin, particularly for distinguishing NDI from other polyuria–polydipsia etiologies.

**Figure 4:**
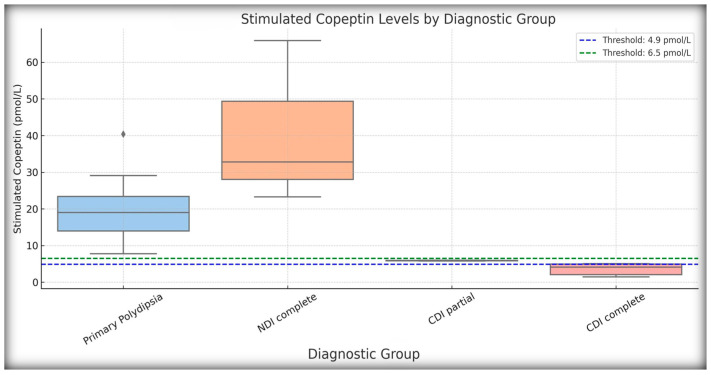
Stimulated copeptin levels by diagnostic group. Boxplot of copeptin concentrations measured after 3% NaCl hypertonic saline infusion. Each box represents the interquartile range (IQR), with the median indicated by a horizontal line and whiskers extending to non-outlier values. Two dashed lines represent key diagnostic thresholds: blue for 4.9 pmol/L and green for 6.5 pmol/L. Patients with NDI (complete forms) showed markedly elevated stimulated copeptin levels (often >30 pmol/L), while patients with complete CDI remained below 4.9, and those with partial CDI remained below 6.5 pmol/L. Those with PP showed normal or elevated values. The figure highlights the physiological separation of diagnostic subtypes based on copeptin response.

These findings validate basal copeptin ≥21.4 pmol/L as a rapid and reliable biomarker for confirming NDI, potentially reducing the need for prolonged testing in clear-cut cases. Boxplots of basal and stimulated copeptin values across the diagnostic subgroups are shown in Figure 3 and Figure 4, respectively.

### 2.5. Agreement Between the WDT and Copeptin Classification

**Figure 5:**
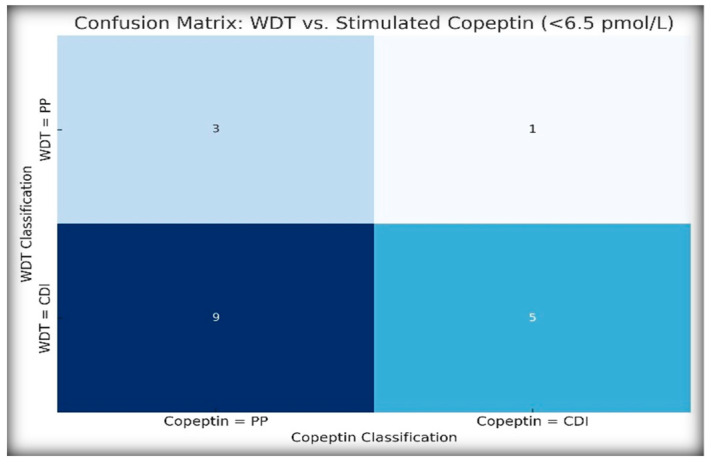
Diagnostic agreement between WDT and copeptin classification. Heatmap-style confusion matrix comparing diagnostic classification between the water deprivation test (WDT) and stimulated copeptin test (<6.5 pmol/L). Rows represent WDT-derived classification, and columns represent the copeptin-derived diagnosis (using <6.5 pmol/L threshold). The WDT classified more patients as having central diabetes insipidus (CDI) than the copeptin test, with statistically significant discordance (McNemar’s *p* = 0.021).

To assess the agreement between traditional and biomarker-based diagnostic strategies, we compared the WDT-based classification (CDI vs. PP) with the stimulated copeptin results using a threshold of <6.5 pmol/L. Figure 5 illustrates diagnostic agreement using a heatmap-style confusion matrix.

Cohen’s κ was 0.063, indicating very weak agreement, while McNemar’s test was statistically significant (*p* = 0.021), confirming that the WDT overdiagnosed CDI in several patients who were later reclassified as PP by copeptin.

In the case of NDI, the WDT classified four patients as having NDI, whereas only three patients met the copeptin criteria for NDI (basal copeptin ≥ 21.4 pmol/L). This discrepancy suggests that copeptin may have a greater specificity, particularly for borderline or atypical presentations.

### 2.6. Feasibility and Telerability Analysuis

An additional analysis was conducted to compare the feasibility, tolerability, and safety of the two diagnostic procedures. The hypertonic saline test had a significantly shorter duration than the water deprivation test (WDT), as confirmed by both the Mann–Whitney U and Wilcoxon signed-rank tests (*p* = 0.0107 and *p* = 0.00178, respectively). Patient tolerance, assessed using a three-point Likert scale, showed a non-significant trend favoring the hypertonic saline test (*p* = 0.071), with fewer cases of moderate or poor tolerance. Importantly, the incidence of adverse effects was significantly higher during the WDT compared to hypertonic saline infusion (OR = 4.86, *p* = 0.0129). The presence of adverse reactions was strongly associated with test duration in both procedures (*p* < 0.0001). Kaplan–Meier’s analysis revealed no significant difference in the timing of adverse reactions between tests (*p* = 0.8271).

### 2.7. Post Hoc Power Analysis

**Figure 6:**
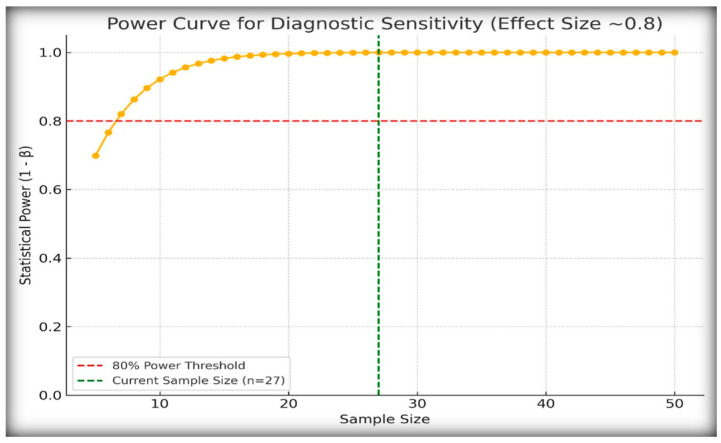
Power curve for post hoc analysis of diagnostic sensitivity. Power curve showing the relationship between sample size and statistical power to detect a diagnostic difference between an observed sensitivity of 100% and a null hypothesis sensitivity of 60% using α = 0.05. The vertical green line marks the actual sample size used in this study (*n* = 27), which achieved a power of 0.999. The red dashed line indicates the conventional 80% power threshold.

Given the rarity of central and nephrogenic diabetes insipidus in the pediatric population, our study included 27 patients with confirmed diagnoses. To assess whether this sample size was adequate for detecting meaningful diagnostic differences, we conducted post hoc power analysis (Figure 6).

We assumed an observed diagnostic sensitivity of 100% for stimulated copeptin at a threshold of <6.5 pmol/L and compared it with the conservative baseline sensitivity of 60%. Using a one-sided hypothesis test with α = 0.05, the calculated power was 0.999, indicating that the study had a 99.9% probability of detecting this effect size.

These findings confirm that, despite the modest cohort size, the diagnostic effect of copeptin was strong enough to yield statistically robust conclusions. This is particularly important in the context of rare endocrine disorders for which large prospective cohorts are often not feasible.

## 3. Discussion

Differential diagnosis of polyuria–polydipsia syndrome (PPS) in pediatric patients remains a clinical challenge, particularly in terms of distinguishing between central diabetes insipidus (CDI), nephrogenic diabetes insipidus (NDI), and primary polydipsia (PP). This study aimed to assess and compare the diagnostic performance of the traditional water deprivation test (WDT) with stimulated copeptin measurement after hypertonic saline infusion using physiologically validated thresholds. We hypothesized that copeptin-based classification would demonstrate superior accuracy, reliability, and feasibility for identifying central diabetes insipidus (CDI), nephrogenic diabetes insipidus (NDI), and primary polydipsia (PP) in pediatric patients.

Our results demonstrate that stimulated copeptin <6.5 pmol/L is a highly accurate marker for CDI, with 100% sensitivity and specificity obtained in our pediatric cohort. These findings are in agreement with those of previous adult and pediatric studies, including large-scale prospective data that established 6.5 pmol/L as a clinically reliable threshold for CDI [5,7,11,13,19]. In contrast, the WDT—despite its long-standing use—showed a weaker agreement with copeptin-based diagnosis (Cohen’s κ = 0.063, and a statistically significant McNemar’s *p* = 0.021) and significantly overclassified CDI in patients later identified as PP based on their copeptin profiles. In our study, three patients initially diagnosed with CDI based on the WDT were reclassified as having PP based on copeptin levels ≥6.5 pmol/L and clinical follow-up. This reclassification underscores the limitations of the WDT in children with partial CDI or functional polydipsia, where incomplete dehydration or variable compliance may obscure diagnostic thresholds. Other pediatric studies, including those by Refardt et al., 2023, and Winzeler et al., 2019, have similarly reported that the WDT tends to overdiagnose CDI in borderline cases, reinforcing the importance of copeptin as a more objective and reproducible marker [3,6,19,20].

ROC curve analysis further confirmed the superiority of copeptin, with an AUC of 1.00 under the <6.5 pmol/L threshold, compared to an AUC of 0.97 for the WDT. While the WDT showed excellent sensitivity (88.9%) and specificity (100%) at a cut-off of ≥14% increase in urine osmolality after DDAVP, it failed to match the diagnostic clarity offered by copeptin. Other authors have similarly shown that copeptin testing is not only more objective but also better aligned with the underlying pathophysiology [2,7,9,21].

Importantly, basal copeptin ≥21.4 pmol/L demonstrated perfect diagnostic performance for identifying NDI, with 100% sensitivity and specificity in confirming three out of four NDI cases initially flagged by the WDT. This is consistent with the recent international consensus guidelines [14] and is supported by pediatric-specific data showing that elevated unstimulated copeptin levels are strongly correlated with AVP resistance [4,9,13]. This diagnostic method allows for early, noninvasive diagnosis in patients in whom dehydration testing may be contraindicated or impractical. In our cohort, three of the four patients diagnosed with NDI by the WDT were confirmed using the basal copeptin threshold, whereas one likely borderline case failed to meet the biochemical criteria. This single patient may reflect methodological overdiagnosis by the WDT—a phenomenon observed in other pediatric cohorts [4,9]. In addition, the clear separation observed in our boxplot analysis between NDI and the other subgroups—with basal copeptin levels exceeding 30 pmol/L in confirmed NDI—provides visual and statistical confirmation of the copeptin value. These findings are in agreement with those of previous studies, including Bockenhauer et al., 2017, and and Christ-Crain et al., 2019, where patients with genetically confirmed NDI exhibited markedly elevated copeptin levels regardless of hydration status [10,22,23]. Our data support the notion that basal copeptin levels can be integrated into the routine first-line screening protocols for suspected NDI in children.

Overall, the findings of this study not only confirm previously published thresholds in a pediatric population but also demonstrate that copeptin testing may prevent diagnostic errors associated with the WDT, particularly in young patients, where test feasibility and accuracy are limited. Beyond diagnostic accuracy, our results also highlight the superior safety and feasibility profile of hypertonic-saline-stimulated copeptin testing. Compared to the water deprivation test, it was shorter in duration, better tolerated, and associated with fewer adverse effects, although the trend for patient-reported tolerance did not reach statistical significance. This adds to the growing body of evidence supporting the incorporation of copeptin as a standard diagnostic biomarker in polyuria–polydipsia syndrome.

### 3.1. Limitations

The study was limited by its single-center design and modest sample size. However, these constraints are mitigated by both the statistical power analysis and consistency of the findings with larger published cohorts. PPS is rare, particularly in pediatric populations, and our post hoc power analysis confirmed that a cohort of 27 patients was sufficiently powered (0.999) to detect significant diagnostic differences, supporting the robustness of our findings. Another potential technical limitation was represented in the WDT protocol using the sublingual formulation of DDAVP instead of the standard IV or intranasal forms. However, this was because of local unavailability, and the dose-adjusted sublingual protocol (Minirin Melt^®^) used in our study has been demonstrated in pharmacodynamic studies to achieve comparable plasma levels and clinical responses, particularly in pediatric patients [24].

Furthermore, NDI diagnosis was based on both copeptin and WDT findings, and it should be taken into consideration that rare genetic subtypes may present with atypical patterns [25,26].

### 3.2. Future Research Directions

Further multicenter studies are needed to validate copeptin thresholds across different age groups, ethnic backgrounds, and clinical scenarios. Additionally, cost-effectiveness analyses comparing copeptin testing to standard protocols could support its broader adoption. Finally, the use of arginine-stimulated copeptin, which may be safer and easier to perform in outpatient settings, warrants further investigation in the pediatric population [12,19].

## 4. Materials and Methods

### 4.1. Study Design and Setting

This prospective, observational, cross-sectional diagnostic accuracy study was conducted at an emergency clinical pediatric hospital in Romania between 2019 and 2025. The study included consecutive pediatric patients referred for evaluation of polyuria–polydipsia syndrome (PPS). The protocol adhered to the STARD (Standards for Reporting Diagnostic Accuracy) guidelines.

### 4.2. Study Population

Eligible participants were children and adolescents aged between 2 and 17 years who presented with persistent polyuria, defined as urine output greater than 2.5 L/m^2^/day or more than 3 mL/kg/h, as well as documented polydipsia, defined as fluid intake exceeding 2 L/m^2^/day [27,28], with or without nocturia. All patients underwent both the standard water deprivation test (WDT) and copeptin measurements following hypertonic saline infusion.

The inclusion criteria for this study were as follows: participants had to be under 18 years of age, present with clinical symptoms of PPS lasting for more than two weeks, and have normal renal function, normal blood glucose levels, and no diagnosis of diabetes mellitus.

The exclusion criteria included the presence of acute illness or dehydration, congenital or acquired renal disease, and current use of medications that affect vasopressin secretion or renal water handling, such as corticosteroids and diuretics. Patients with incomplete clinical data or whose parents did not provide informed consent were also excluded from the study.

Ethical approval for initiating the study was granted by the Medical Ethics Committee of the “Sf. Ioan” Children’s Emergency Hospital in Galati, Romania, under approval number 6257/01.10.2019, prior to the enrollment of any patient. All the participants were pediatric patients admitted to this hospital for the specific diagnostic procedures used in this study. The approval explicitly covered both clinical data collection and the publication of anonymized results. Informed written consent was obtained from the parents or legal guardians of all participants, including specific agreement for the use of anonymized data in scientific publications. All procedures complied with the ethical standards of the institutional research board, the Declaration of Helsinki (revised in 2013), and current data protection regulations, including the General Data Protection Regulation (GDPR). An additional ethics approval (reference no. C5531/18.03.2025) was issued by the same institution to authorize extended data analysis and publication of the final results that are presented in this article. Both approvals reflect full institutional compliance with national and international guidelines for research involving minors and clinical data protection.

### 4.3. Diagnostic Procedures

#### 4.3.1. The Water Deprivation Test (WDT) with Desmopressin (DDAVP)

The WDT was conducted according to standard pediatric protocols. The hydration status was standardized before starting the WDT, and patients were allowed to consume liquids ad libitum. The restriction phase started in the morning of the test, at 8:00 a.m., while the patients were monitored under medical supervision with hourly weight, serum sodium, serum osmolality, and urine osmolality measurements.

The test was terminated upon reaching one of the following predefined clinical endpoints: a decrease in body weight of at least 3%, a serum osmolality exceeding 295–300 mOsm/kg, or the onset of clinical symptoms such as fatigue, irritability, or hypotension [3].

After reaching one of these predefined endpoints, desmopressin acetate—a synthetic analog of 1-deamino-8-D-arginine vasopressin (DDAVP)—was administered using the sublingual formulation Minirin Melt^®^ due to the unavailability of intravenous or intranasal formulations in Romania at the start of the study [7].

The dose of DDAVP was adjusted according to age and body weight. Children older than 12 years of age received 240 μg sublingually, a dose shown to achieve serum concentrations comparable to 2 μg intravenous DDAVP. Children aged between 2 and 12 years received 120 μg sublingually, equivalent to 1 μg administered intravenously [13,23,24].

Although oral formulations of desmopressin generally have lower bioavailability, the sublingual lyophilizate (wafer) used in this study offers more predictable absorption, faster onset, and improved tolerability compared to standard oral tablets. Previous pharmacokinetic studies have demonstrated comparable plasma concentrations and clinical efficacy of sublingual desmopressin compared to intravenous formulations, supporting its use in clinical diagnostics [24].

The final urine osmolality was measured 1–2 h post-DDAVP administration. A ≥50% increase in urine osmolality was considered diagnostic for CDI, whereas a final urine osmolality >800 mOsm/kg was used to identify primary polydipsia (PP).

#### 4.3.2. Hypertonic Saline Infusion and Stimulated Copeptin Measurement

Hypertonic saline (3% NaCl) was administered intravenously, starting with a bolus volume of 250 mL, followed by a continuous infusion at a rate of 0.15 mL/kg/min. Clinical and laboratory parameters were continuously monitored throughout the procedure. The infusion was continued until either the serum sodium level reached ≥150 mmol/L or the serum osmolality was ≥300 mOsm/kg.

Blood samples for copeptin were collected at two time points: at baseline (prior to infusion) and at the endpoint of the stimulation protocol. Copeptin concentrations were measured using a standardized fluorescence immunoassay (FIA), performed using the BRAHMS LIA platform (Thermo Fisher Scientific, Hennigsdorf, Germany). This method has demonstrated an excellent correlation with the widely used BRAHMS Kryptor Compact Plus chemiluminescence assay, as validated in large-scale diagnostic studies [29]. To ensure optimal copeptin stability, blood samples were collected in chilled EDTA tubes, immediately centrifuged at 4 °C, and stored at −80 °C until analysis.

The diagnostic thresholds applied in this study were consistent with previously validated cut-off values. A stimulated copeptin concentration of <4.9 pmol/L was considered suggestive of complete central diabetes insipidus (CDI), while a value of <6.5 pmol/L indicated either complete or partial forms of CDI. Basal (unstimulated) copeptin concentration ≥21.4 pmol/L is considered highly indicative of nephrogenic diabetes insipidus (NDI). Stimulated values falling between 6.5 and 21.4 pmol/L were interpreted as suggestive of primary polydipsia (PP) or partial forms of CDI or NDI, depending on the clinical context and accompanying biochemical profile.

The sodium levels, as well as the rest of the biological parameters used in this study, were measured using the Ortho Vitros 4600 chemistry system and Ortho Vitros 5,1FS Chemistry Analyzer (Ortho Clinical Diagnostics, Raritan, NJ, USA) using the same technical method.

### 4.4. Outcome Measures

The primary outcome of this study was the diagnostic performance of stimulated copeptin in comparison with the water deprivation test (WDT) for differentiating central diabetes insipidus (CDI) from primary polydipsia (PP). Diagnostic performance was assessed using standard accuracy metrics, including sensitivity, specificity, positive predictive value (PPV), negative predictive value (NPV), receiver operating characteristic (ROC) curves, and area under the curve (AUC).

The secondary outcomes included the feasibility of each diagnostic method, which was evaluated by measuring the test duration and occurrence of adverse effects. Additionally, patient tolerance was assessed using a 3-point Likert scale, where a score of 1 indicated good tolerance, 2 indicated moderate tolerance, and 3 indicated poor tolerance. Another secondary objective was to evaluate the diagnostic accuracy of basal copeptin in identifying nephrogenic diabetes insipidus (NDI), based on previously validated threshold values.

### 4.5. Statistical Analysis

All statistical analyses were performed using Python 3.14 (with SciPy, Pandas, and Matplotlib libraries) within the IDLE environment. Advanced AI language models helped us in code generation to enhance precision and efficiency, and each step of the process was reviewed by experts to guarantee validity. In addition, key analyses were cross-checked using the RStudio software (R version 4.4.1 (14 June 2024)) or IBM SPSS (version 29) to ensure methodological rigor and consistency of the findings derived from Python scripts. Continuous variables are reported as mean ± standard deviation (SD) or median (interquartile range [IQR]), while categorical variables are expressed as frequencies and percentages.

Diagnostic accuracy was assessed by calculating the sensitivity, specificity, positive/negative predictive values, likelihood ratios (LR+, LR−), receiver operating characteristic (ROC) curve, and area under the curve (AUC). Agreement between diagnostic tests (WDT vs. copeptin) was evaluated using Cohen’s kappa coefficient and McNemar’s test.

For the additional feasibility and tolerability analysis, non-parametric tests were applied because of non-normal distribution of variables (as determined by the Shapiro–Wilk test). The Mann–Whitney U test and the Wilcoxon signed-rank test were used to compare the duration and tolerability scores between diagnostic procedures. Fisher’s exact test was used to compare the incidence of adverse events. Kaplan–Meier curves and the log-rank test were used to analyze the time to the onset of adverse reactions.

A post hoc power analysis was performed to confirm the adequacy of the sample size based on the observed diagnostic sensitivity. A *p*-value < 0.05 was considered statistically significant throughout the analysis.

## 5. Conclusions

Our study demonstrated that copeptin measurement after hypertonic saline infusion is a highly accurate and feasible diagnostic tool for distinguishing central diabetes insipidus (CDI) from primary polydipsia (PP) in pediatric patients presenting with polyuria–polydipsia syndrome (PPS). At a copeptin threshold of <6.5 pmol/L, the test showed perfect diagnostic performance (100% sensitivity and specificity), clearly surpassing the traditional water deprivation test (WDT) in terms of specificity, diagnostic clarity, and patient tolerability. Additionally, basal copeptin concentrations ≥21.4 pmol/L provided a reliable and noninvasive biomarker for nephrogenic diabetes insipidus (NDI), correctly identifying all confirmed cases in this study.

Given these findings, copeptin testing, both basal and stimulated, has emerged as a superior alternative to the conventional WDT, offering substantial clinical advantages such as a shorter test duration, greater objectivity, reduced patient discomfort, and improved diagnostic reliability. We propose that copeptin measurements, particularly those that stimulate copeptin after hypertonic saline infusion, should be considered as a first-line diagnostic approach for pediatric patients with PPS, especially in cases where WDT results are equivocal or practically difficult to interpret.

Future multicenter studies, along with cost-effectiveness analyses, are necessary to further validate these thresholds across broader pediatric populations, including different age groups and clinical scenarios, thus facilitating wider clinical adoption and standardized protocols.

## Figures and Tables

**Table 1:** Diagnostic performance metrics for the WDT.

Diagnostic Threshold ^1^ (% Osm Increase)	Sensitivity	Specificity	* PPV	* NPV	* AUC
≥14%	88.9% (67–97%)	100% (70–100%)	100% (81–100%)	81.8% (52–95%)	0.972

^1^ Table 1 summarizes the accuracy of the water deprivation test for diagnosing central diabetes insipidus (CDI) at the optimal cut-off. * PPV—positive predictive value; * NPV—negative predictive value; * AUC—area under the curve.

**Table 2:** Diagnostic accuracy of stimulated copeptin for CDI vs. PP.

Diagnostic Threshold ^1^	Sensitivity	Specificity	* PPV	* NPV	* AUC
4.9 pmol/L	40%	100%	100%	68.4%	0.73
6.5 pmol/L	100%	100%	100%	100%	1.0

^1^ Table 2 summarizes the diagnostic performance metrics for each copeptin threshold. * PPV—positive predictive value; * NPV—negative predictive value; * AUC—area under the curve.

## Data Availability

Personal medical data are publicly unavailable due to privacy or ethical restrictions, being obtained from the medical record of the patient admitted into the Emergency Clinical Hospital for Children Sf Ioan Galati.

## Abbreviations

The following abbreviations are used in this manuscript:

PPS	Polyuria–Polydipsia Syndrome
CDI	Central Diabetes Insipidus
NDI	Nephrogenic Diabetes Insipidus
PP	Primary Polydipsia
WDT	Water Deprivation Test
DDAVP	1-deamino-8-D-arginine vasopressin (Desmopressin Acetate)
ADH	Antidiuretic Hormone
AVP	Arginine Vasopressin
ROC	Receiver Operating Characteristic
AUC	Area Under the Curve
PPV	Positive Predictive Value
NPV	Negative Predictive Value
LR	Likelihood Ratio
STARD	Standards for Reporting Diagnostic Accuracy
MIS-C	Multisystem Inflammatory Syndrome in Children
FIA	Fluorescence Immunoassay
IQR	Interquartile Range
SD	Standard Deviation
SPSS	Statistical Package for the Social Sciences
LIA	Luminescence Immunoassay
